# NDH-1 Is Important for Photosystem I Function of *Synechocystis* sp. Strain PCC 6803 under Environmental Stress Conditions

**DOI:** 10.3389/fpls.2017.02183

**Published:** 2018-01-17

**Authors:** Jiaohong Zhao, Fudan Gao, Da-Yong Fan, Wah Soon Chow, Weimin Ma

**Affiliations:** ^1^Department of Biology, College of Life and Environment Sciences, Shanghai Normal University, Shanghai, China; ^2^State Key Laboratory of Vegetation and Environmental Change, Institute of Botany, Chinese Academy of Sciences, Beijing, China; ^3^Division of Plant Sciences, Research School of Biology, Australian National University, Canberra, ACT, Australia

**Keywords:** NDH-1, NDH-1-PSI, PSI function, environmental stress, *Synechocystis* sp. strain PCC 6803

## Abstract

Cyanobacterial NDH-1 interacts with photosystem I (PSI) to form an NDH-1-PSI supercomplex. Here, we observed that absence of NDH-1 had little, if any, effect on the functional fractions of PSI under growth conditions, but significantly reduced the functional fractions of PSI when cells of *Synechocystis* sp. strain PCC 6803 were moved to conditions of multiple stresses. The significant reduction in NDH-1-dependent functional fraction of PSI was initiated after PSII activity was impaired. This finding is consistent with our observation that the functional fraction of PSI under growth conditions was rapidly and significantly decreased with increasing concentrations of DCMU, which rapidly and significantly suppressed PSII activity by blocking the transfer of electrons from *Q*_A_ to *Q*_B_ in the PSII reaction center. Furthermore, absence of NDH-1 resulted in the PSI limitation at the functionality of PSI itself but not its donor-side and acceptor-side under conditions of multiple stresses. This was supported by the result of a significant destabilization of the PSI complex in the absence of NDH-1 but the presence of multiple stresses. Based on the above results, we propose that NDH-1 is important for PSI function of *Synechocystis* sp. strain PCC 6803 mainly via maintaining stabilization of PSI under conditions of environmental stresses.

## Introduction

Cyanobacterial NDH-1 is predominantly, if not totally, located in the thylakoid membrane (Ohkawa et al., 2001, 2002; Zhang et al., 2004; Xu et al., 2008; Battchikova et al., 2011), and participates in a variety of bioenergetic reactions, including respiration, cyclic electron transport around PSI, and CO_2_ acquisition (Ogawa, 1991; Mi et al., 1992; Ohkawa et al., 2000). The function of NDH-1 is usually minor under normal growth conditions and becomes important under conditions of environmental stresses. In line with this, absence of NDH-1 has little, if any, effect on cell growth under normal conditions but retards cell growth and even causes cell death under conditions of environmental stresses, such as high light (Battchikova et al., 2011; Dai et al., 2013; Zhang et al., 2014; Zhao et al., 2014b, 2015; Gao et al., 2016a; Wang et al., 2016) and high temperature (Zhao et al., 2014a; Gao et al., 2016b). Therefore, cyanobacterial NDH-1 plays an important role in coping with various environmental stresses.

When cyanobacterial cells are transferred from normal growth conditions to multiple stressful environments, the amount of NDH-1 and its activity, for example, NDH-1-dependent cyclic electron transport around PSI (NDH-CET), are significantly increased under conditions of high temperature (Rowland et al., 2010; Zhao et al., 2014a), high salt (Hibino et al., 1996; Tanaka et al., 1997), high light (Mi et al., 2001), and low CO_2_ (Deng et al., 2003). Such an increase is assumed to be important in repairing the photodamaged PSII, optimizing photosynthesis by increasing the proton gradient across the thylakoid membrane and supplying additional ATP (Allakhverdiev et al., 2005). However, the effect of cyanobacterial NDH-1 on PSI remains elusive.

Recently, cyanobacterial NDH-1 was found to interact with PSI to form an NDH-1-PSI supercomplex via CpcG2-phycobilisome, a PSI-specific antenna (Kondo et al., 2007; Gao et al., 2016a). Further, the supercomplex was found to be involved in NDH-CET but not respiration and CO_2_ uptake (Gao et al., 2016a). Here, our results demonstrate that NDH-1 is important for PSI function of *Synechocystis* sp. strain PCC 6803 (hereafter *Synechocystis* 6803) under conditions of multiple stresses. We further found that NDH-1-dependent PSI function was initiated after PSII was impaired and its impairment is mostly the result of destabilization of PSI. The contribution of NDH-1-PSI supercomplex to PSI function is discussed.

## Materials and methods

### Culture conditions

A glucose tolerant strain of wild-type (WT) *Synechocystis* 6803 and its mutants, Δ*ndhB* (M55) (Ogawa, 1991), M55+NdhB and Δ*pgr5* were cultured at 30°C in BG-11 medium (Allen, 1968) buffered with Tris-HCl (5 mM, pH 8.0) by bubbling with 2% (*v*/*v*) CO_2_ in air. Continuous illumination was provided by fluorescence lamps at 40 μmol photons m^−2^s^−1^. The mutant strains were grown in the presence of appropriate antibiotics.

### Construction of Δ*pgr5* mutant

The Δ*pgr5* mutant was constructed as follows. The upstream and downstream regions of *ssr2016* (*pgr5*) were amplified by PCR, creating appropriate restriction sites. A DNA fragment encoding a kanamycin resistance (Kam^R^) cassette was also amplified by PCR, creating *Sal*I and *Xba*I sites using appropriate PCR primers, *pgr5*-C and *pgr5*-D (Supplementary Table S1). These three PCR products were ligated into the multiple cloning site of pUC19 (**Figure 5A**) and used to transform the WT cells of *Synechocystis* 6803 to generate the Δ*pgr5* mutant. The transformants were spread on agar plates containing BG-11 medium and kanamycin (10 μg mL^−1^) buffered at pH 8.0, and the plates were incubated in 2% (*v*/*v*) CO_2_ in air under illumination by fluorescent lamps at 40 μmol photons m^−2^ s^−1^ as described previously (Williams and Szalay, 1983; Long et al., 2011). The mutated *pgr5* in the transformants was segregated to homogeneity (by successive streak purification) as determined by PCR amplification and reverse transcription (RT)-PCR analysis (**Figures 5B,C**).

### Construction of M55 complementation strain

The M55 complementation strain was constructed as follows. A DNA fragment containing the *ndhB* gene was amplified by PCR and then inserted into *Nde*I site of P*psbAII* expression vector (Jiang et al., 2012) to form the P*psbAII*-*ndhB* expression plasmid (Supplementary Figure S1A; primers are shown in Supplementary Table S1), which was used to transform the M55 mutant of *Synechocystis* 6803 to generate the M55 complementation strain. The transformants were spread on agar plates containing BG-11 medium, spectinomycin (10 μg mL^−1^) and kanamycin (10 μg mL^−1^) buffered at pH 8.0, and the plates were incubated in 2% (*v*/*v*) CO_2_ in air under illumination by fluorescent lamps at 40 μmol photons m^−2^ s^−1^ as described previously (Williams and Szalay, 1983; Long et al., 2011). Complete segregation of the transformants was confirmed by PCR (Supplementary Figure S1B) and the expression level of *ndhB* in the transformants was estimated by RT-PCR (Supplementary Figure S1C).

**Figure 1:**
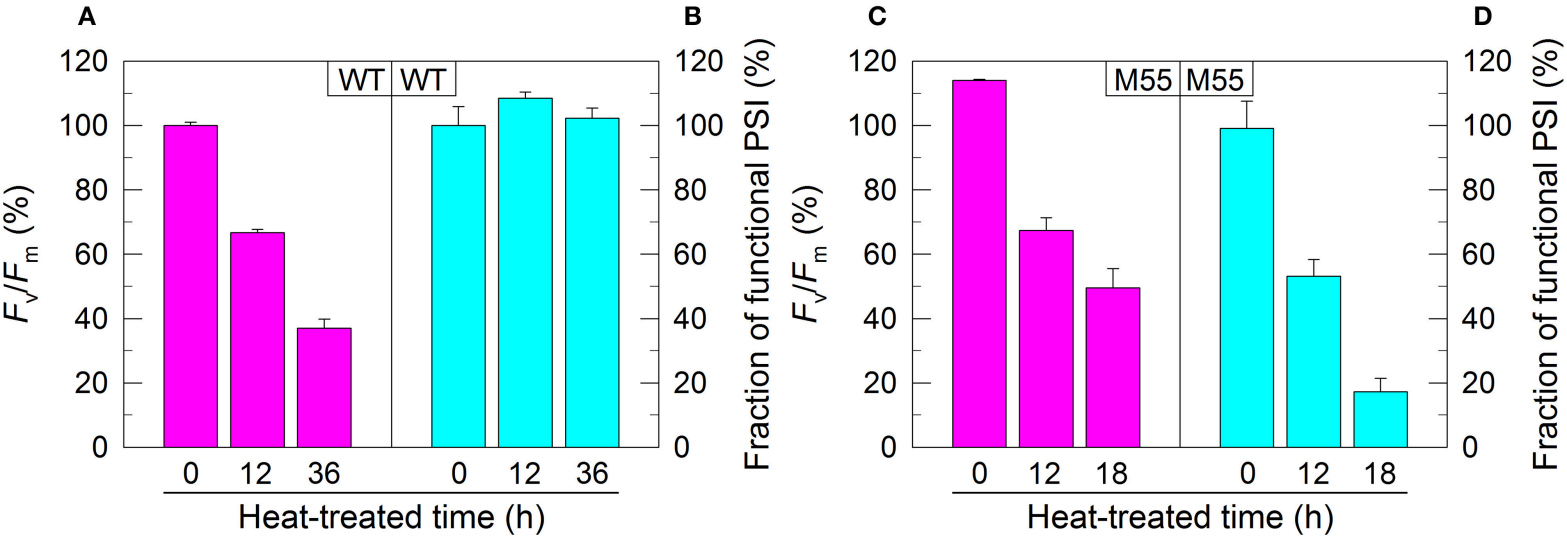
PSII activity and PSI functional fraction of WT **(A,B)** and M55 **(C,D)** under high temperature conditions. Cells were grown at 30°C for 24 h and were transferred to 45°C for different periods. Prior to the measurements, the Chl *a* concentration was adjusted to 20 μg mL^−1^. PSII activity and PSI functionality were determined by the *F*_v_/*F*_m_ and *P*_m_ parameters, respectively, expressed as percentage of the WT (100%). The *F*_v_/*F*_m_ and *P*_m_ values that correspond to 100% are 0.46 ± 0.02 and 0.58 ± 0.02, respectively. Values are means ± *SD* (*n* = 5).

### RNA extraction and RT-PCR analysis

Total RNA was isolated and analyzed as described previously (McGinn et al., 2003). RT-PCR was performed using the Access RT-PCR system (Promega) to generate products corresponding to *pgr5* and *16 S rRNA*, with 0.5 μg of DNase-treated total RNA as starting material. RT-PCR conditions were 95°C for 5 min followed by cycles of 95, 62, and 72°C for 30 s each. The reactions were stopped after 25 cycles for *16 S rRNA* and after 35 cycles for *ndhB* and *pgr5*. The primers used are summarized in Supplementary Table S1.

### Chlorophyll fluorescence

The yield of chlorophyll (Chl) fluorescence at the steady state of electron transport was measured using a Dual-PAM-100 monitoring system (Walz, Effeltrich, Germany) equipped with an ED-101US/MD unit (Schreiber et al., 1986; Ma et al., 2008), as shown in Supplementary Figure S2. Minimal fluorescence corresponding to open PSII centers in the dark-adapted state (*F*_o_) and in the far-red (FR) light-adapted state (*F*_o_′) was excited by a weak measuring light (650 nm) at a PFD of 0.05–0.15 μmol photons m^−2^s^−1^. A saturation pulse of red light (100 ms, 10,000 μmol photons m^−2^s^−1^) was applied to determine the maximal fluorescence at closed PSII centers in the dark-adapted state (*F*_m_) and in the red actinic light (AL)-adapted state (*F*_m_′) as described previously (Klughammer and Schreiber, 2008a). Subsequently, red AL was applied to monitor fluorescence under the steady-state condition (*F*_s_). *F*_v_/*F*_m_ and *q*P were calculated as (*F*_m_-*F*_o_)/*F*_m_ (Kitajima and Butler, 1975) and (*F*_m_′-*F*_s_)/(*F*_m_′-*F*_o_′) (van Kooten and Snel, 1990), respectively.

### The P700^+^ signal monitored as an 830 nm absorbance change

With the Dual-PAM-100, P700^+^ was monitored as the absorption difference between 830 and 875 nm in transmission mode. The quantum yields of PSI were determined using the saturation pulse method as described previously (Klughammer and Schreiber, 1994, 2008b; Supplementary Figure S3). The level of maximal P700^+^ signal observed upon full oxidation, *P*_m_, was determined by application of a saturation pulse of red light (100 ms; 10,000 μmol photons m^−2^s^−1^) in the presence of far-red light (about 0.3 μmol photons m^−2^s^−1^) at 720 nm.

### Photosynthetic oxygen evolution

Oxygen evolution by photosynthesis was measured at 30°C in the presence of 10 mM NaHCO_3_ with a Clark-type oxygen electrode (Hansatech, Hertfordshire, UK) according to the method described by Ma and Mi (2005). Cells were suspended in fresh BG-11 medium at a Chl *a* concentration of 5 μg mL^−1^ and, different concentrations of 3-(3,4-dichlorophenyl)-1,1-dimethylurea (DCMU) were added to the cell suspension cultures prior to measurement. The intensity of AL used for the measurement of photosynthetic oxygen evolution was 800 μmol photons m^−2^ s^−1^.

### Isolation of crude thylakoid membranes

Cell in cultured medium (800 mL) were harvested at the logarithmic phase of growth and washed twice by suspending in 50 mL of fresh BG-11 medium, and the thylakoid membranes were isolated according to Gombos et al. (1994) with some modifications as follows. The cells suspended in 5 mL of disruption buffer (10 mM HEPES-NaOH, 5 mM sodium phosphate, pH 7.5, 10 mM MgCl_2_, 10 mM NaCl, and 25% [*v*/*v*] glycerol) were supplemented by zirconia/silica beads and broken by vortexing 20 times at the highest speed for 30 s at 4°C with 5 min of cooling on ice between the runs. The crude extract was centrifuged at 5,000×*g* for 5 min to remove the glass beads and unbroken cells. By further centrifugation at 20,000×*g* for 30 min, we obtained crude thylakoid membranes as precipitates.

### Electrophoresis

Blue-native (BN)-PAGE of *Synechocystis* 6803 membranes was performed as described previously (Kügler et al., 1997) with slight modifications (Battchikova et al., 2011; Dai et al., 2013; Zhang et al., 2014; Zhao et al., 2014a, 2015; Gao et al., 2016a,b; Wang et al., 2016). Isolated membranes were prepared for BN-PAGE as follows. Membranes were washed with 330 mM sorbitol, 50 mM Bis-Tris (pH 7.0), and 0.5 mM phenylmethylsulfonyl fluoride (PMSF; Sigma) and resuspended in 20% (*w*/*v*) glycerol, 25 mM Bis-Tris (pH 7.0), 10 mM MgCl_2_, 0.1 units of RNase-free DNase RQ1 (Promega) at a Chl *a* concentration of 0.25 mg mL^−1^, and 0.5 mM PMSF. The samples were incubated on ice for 10 min, and an equal volume of 3% DM was added. Solubilization was performed for 40 min on ice. Insoluble components were removed by centrifugation at 18,000 × *g* for 15 min. The collected supernatant was mixed with one-tenth volume of sample buffer, 5% Serva Blue G, 100 mM Bis-Tris (pH 7.0), 30% (*w*/*v*) sucrose, 500 mM ε-amino-*n*-caproic acid, and 10 mM EDTA. Solubilized membranes were then applied to a 0.75-mm-thick, 5–12.5% acrylamide gradient gel (Hoefer Mighty Small mini-vertical unit). Samples were loaded on an equal Chl *a* basis per lane. Electrophoresis was performed at 4°C by increasing the voltage gradually from 50 up to 200 V during the 5.5-h run.

## Results

### NDH-1 is important for PSI function under conditions of multiple stresses

In *Synechocystis* 6803, NDH-1 interacts with PSI to form an NDH-1-PSI supercomplex (Gao et al., 2016a). To test whether the absence of NDH-1 affected the function of PSI under conditions of growth and environmental stress, we monitored the fractions of functional PSI when cells of WT and Δ*ndhB* (M55), which lacks all functional NDH-1 complexes (Zhang et al., 2004), were incubated under high temperature conditions for different periods. The fractions of functional PSI of WT and M55 were similar under conditions of growth temperature (30°C), as deduced from the maximal P700 change (*P*_m_) level (0 h in Figures 1B,D). When cells were moved to conditions of high temperature (45°C) for different periods, however, we observed some expected and unexpected results. As expected, the activity of PSII in WT and M55 was gradually decreased (Figures 1A,C) whereas the fraction of functional PSI in WT was always maintained at a high level (Figure 1B) with the extension of heating time. Unexpectedly, the fraction of functional PSI in M55 was gradually and drastically decreased with the increase in heat incubation (Figure 1D).

**Figure 2:**
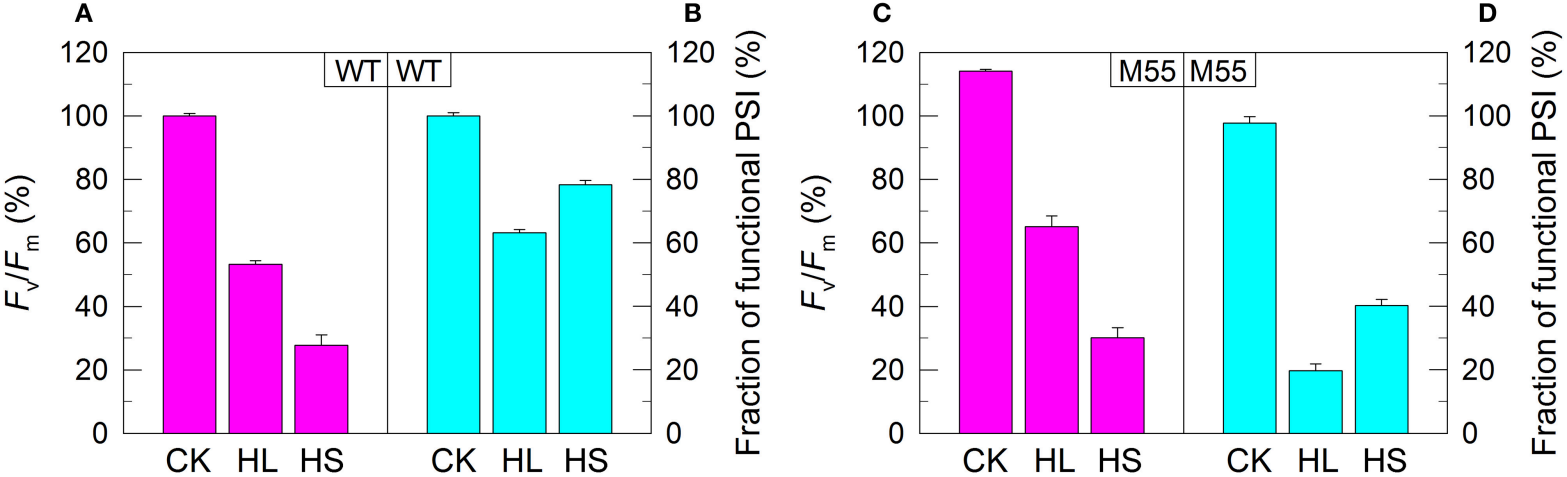
PSII activity and PSI functional fraction of WT **(A,B)** and M55 **(C,D)** under conditions of high light and high salt. Cells were grown under normal salt conditions at 40 μmol photons m^−2^ s^−1^ for 24 h and were transferred to 300 μmol photons m^−2^ s^−1^ for 36 h or to 0.8 M NaCl for 12 h. Prior to the measurements, the Chl *a* concentration was adjusted to 20 μg mL^−1^. PSII activity and PSI functionality were determined by the *F*_v_/*F*_m_ and *P*_m_ parameters, respectively, expressed as percentage of the WT (100%). The *F*_v_/*F*_m_ and *P*_m_ values that correspond to 100% are shown in the legend of Figure 1. Values are means ± *SD* (*n* = 5). CK, control check; HL, high light; HS, high salt.

We also measured the fractions of functional PSI upon exposure of WT and M55 cells to conditions of high light and high salt. As expected, the remaining functional fraction of PSI in M55 was about 20% of that in the WT under high light and was about 40% of that in the WT under high salt (Figures 2B,D). It is worthy of note that these decreased fractions of functional PSI in M55 under conditions of heat, high light, and high salt could be recovered in its complementation strain M55+NdhB to the levels of WT (Supplementary Figures S1D–F). Taking all these results together, we can conclude that NDH-1 is important for the function of PSI under conditions of environmental stresses but not under growth conditions.

### NDH-1-dependent PSI function is initiated after PSII activity is impaired

Under conditions of normal growth, WT and M55 cells had a similar activity of PSII (measured as the maximal quantum yield of PSII, *F*_v_/*F*_m_) and had also a similar functional activity of PSI (0 h in Figures 1A–D). When cells of WT and M55 were transferred to conditions of multiple stresses and the activity of PSII was significantly suppressed in both strains, the functional fraction of PSI was significantly decreased in M55 mutant but not (Figure 1B) or to a lesser extent (Figure 2B) in the WT. It appears plausible that the decrease in PSII activity is a prerequisite for initiating the NDH-1-depenent PSI function.

**Figure 3:**
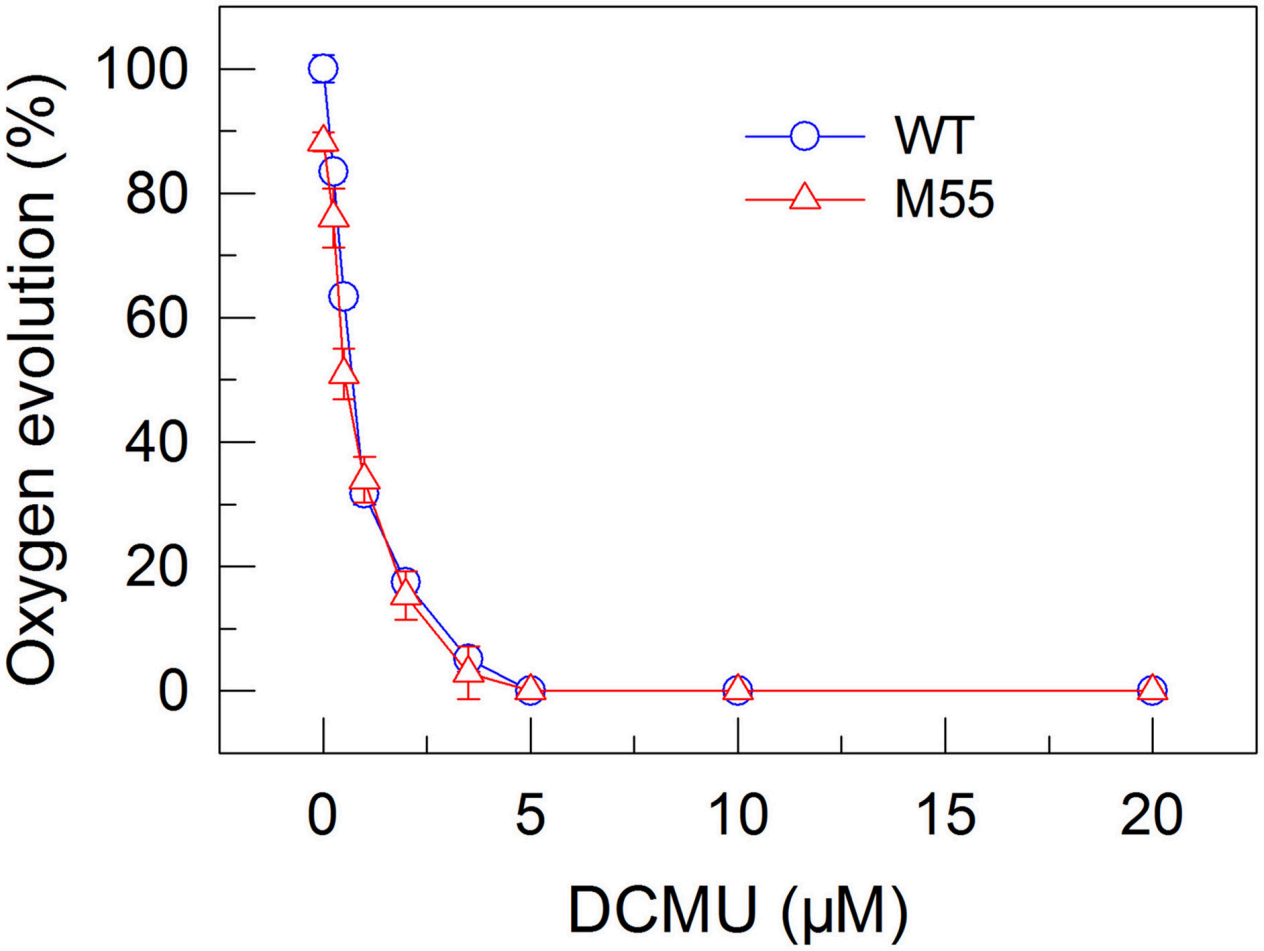
Effects of DCMU treatment at different concentrations on the PSII activity in WT and M55 strains. PSII activity was assessed by the light-saturated photosynthetic oxygen evolution rate in the presence of DCMU and NaHCO_3_, expressed as percentage of the WT (100%). The rate of photosynthetic oxygen evolution that corresponds to 100% is 128.2 ± 8.6 μmol O_2_ mg^−1^ Chl *a* h^−1^. Values are means ± *SD* (*n* = 5).

**Figure 4:**
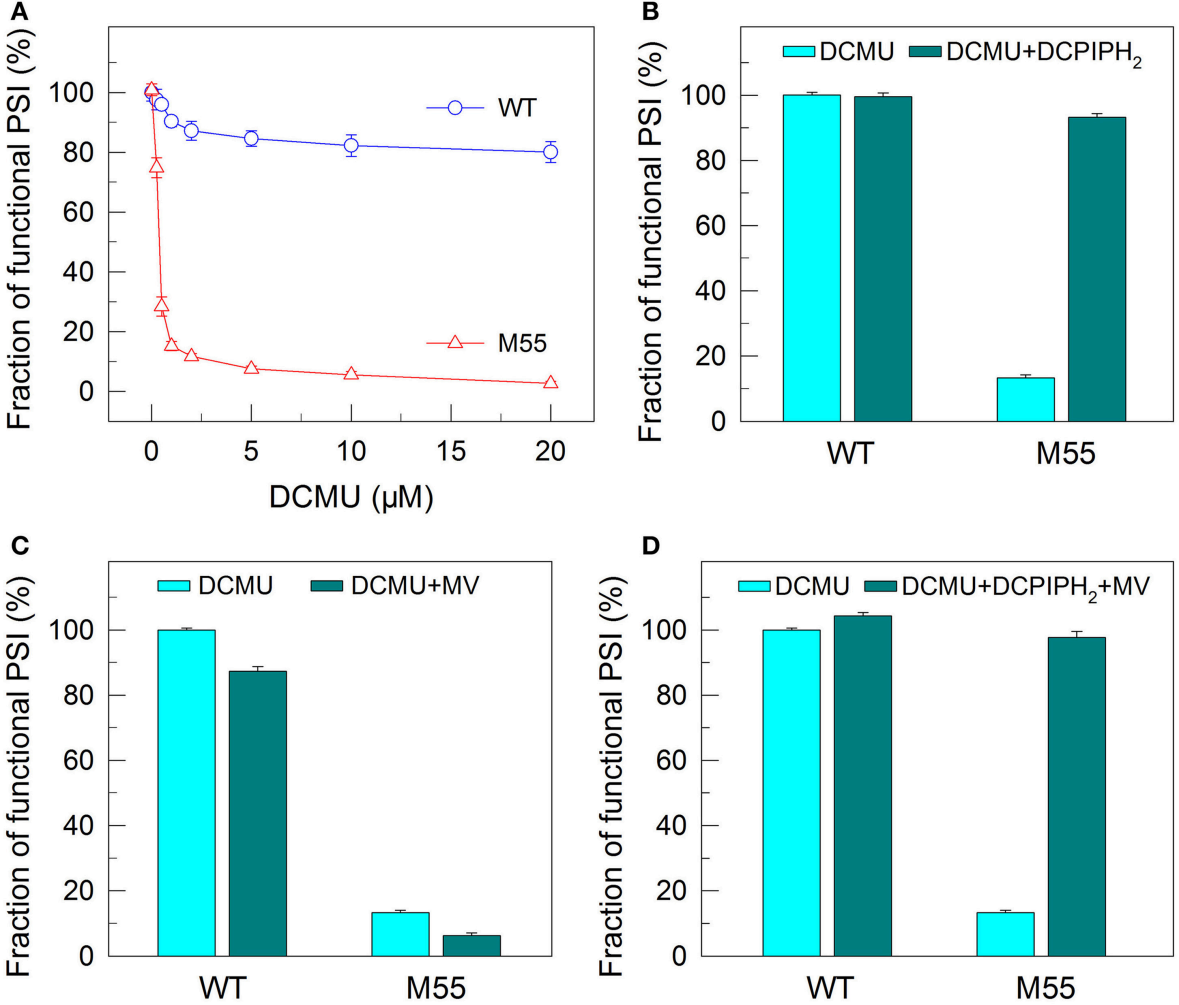
Functional fractions of PSI in WT and M55 with or without PSII electron transport inhibitor and/or PSI electron donor and acceptor. Cells were grown under 2% CO_2_ at 40 μmol photons m^−2^ s^−1^ and were collected during their logarithmic growth phase. The Chl *a* concentration of WT and M55 cells was adjusted to 20 μg mL^−1^. Prior to the *P*_m_ measurements, DCMU of different concentrations **(A)** or 10 μM **(B–D)** was added to the cell suspension **(A)**, to the cell suspension containing 200 μM 2,6-dichlorophenol-indophenol and 5 mM sodium ascorbate **(B)**, to the cell suspension containing 200 μM methyl viologen **(C)**, and to the cell suspension containing 200 μM 2,6-dichlorophenol-indophenol, 5 mM sodium ascorbate, and 200 μM methyl viologen **(D)**. The functionality of PSI reaction centers was determined by the *P*_m_ parameter, expressed as a percentage of the WT (100%). The *P*_m_ value that corresponds to 100% is shown in the legend of Figure 1. Values are means ± *SD* (*n* = 5).

**Figure 5:**
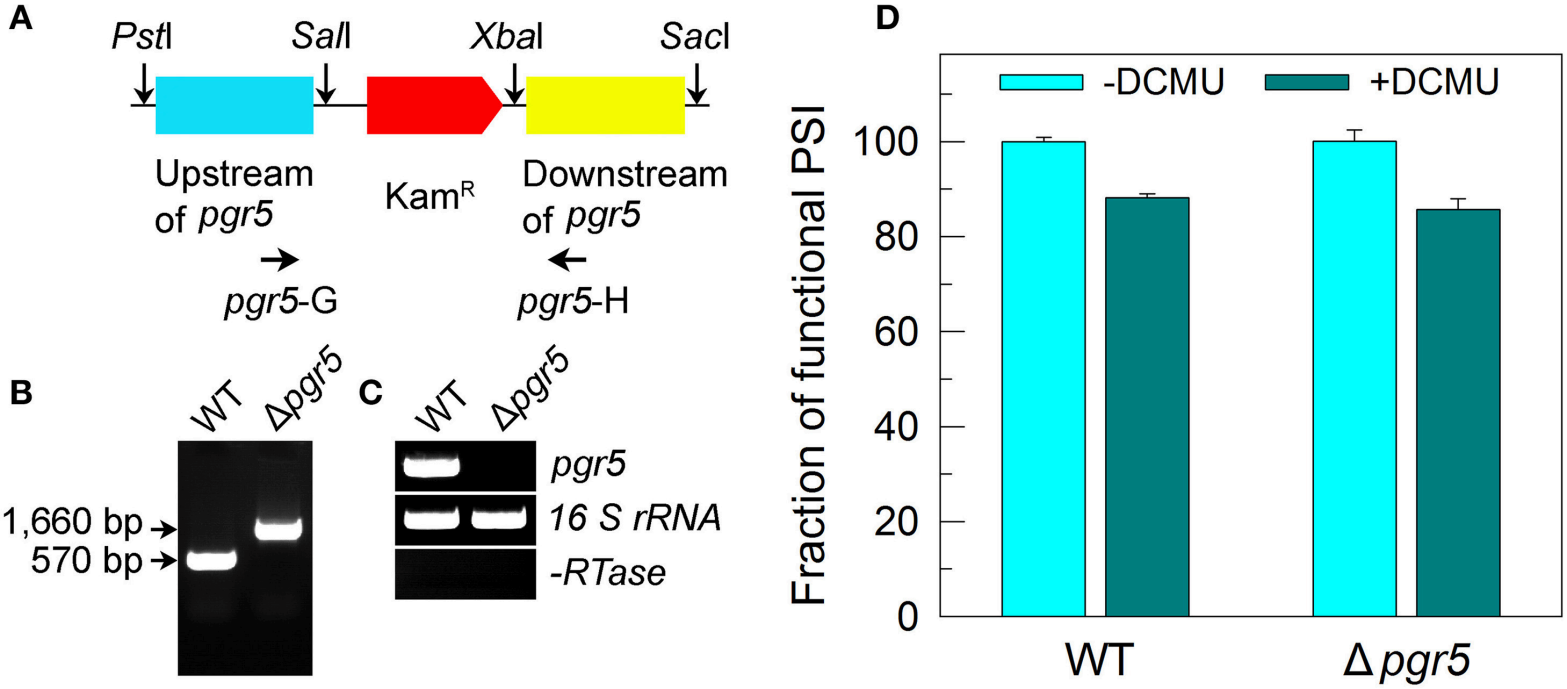
Deletion of *pgr5* and its effect on *P*_m_ in the presence of DCMU. **(A)** Construction of the plasmid used to generate the *pgr5* deletion mutant (Δ*pgr5*). **(B)** PCR segregation analysis of the Δ*pgr5* mutant using the *pgr5*-G and *pgr5*-H primer sequences (Supplementary Table S1). **(C)** Transcript levels of *pgr5* in the WT and Δ*pgr5* strains. The transcript level of *16 S rRNA* in each sample is shown as a control. The absence of DNA contamination was confirmed by PCR without reverse transcriptase reaction. **(D)** Cells were grown under 2% CO_2_ at 40 μmol photons m^−2^ s^−1^ and were collected during their logarithmic growth phase. The Chl *a* concentration of WT and Δ*pgr5* cells was adjusted to 20 μg mL^−1^. Prior to the *P*_m_ measurements, DCMU (10 μM; final concentration) was added to the cell suspension. The functionality of PSI reaction centers was determined by the *P*_m_ parameter, expressed as a percentage of the WT (100%). The *P*_m_ value that corresponds to 100% is shown in the legend of Figure 1. Values are means ± *SD* (*n* = 5).

To confirm this possibility, we measured the fractions of functional PSI in WT and M55 cells without or with different concentrations of DCMU, which blocks the transfer of electrons from *Q*_A_ to *Q*_B_ in the PSII reaction center (Trebst, 1980). As expected, with the increase in DCMU concentration, the activity of PSII (measured as oxygen evolution) was rapidly and similarly suppressed in WT and M55 (Figure 3). By contrast, the fractions of functional PSI were rapidly and significantly decreased in M55 but much less in the WT with the increase in DCMU concentration (Figure 4A), although both strains had a similar function of PSI in the absence of DCMU (0 μM in Figure 4A). Under conditions of DCMU addition, however, these changes in PSI function in the absence of NDH-1 were not observed in the absence of PROTON GRADIENT REGULATION 5 (PGR5) (Figures 5A–D), an alternative to NDH-1 in cyclic electron transport around PSI (Yeremenko et al., 2005). Based on the above results, we can conclude that NDH-1-depenent PSI function is specifically initiated after PSII activity is impaired but PGR5 is not involved in this process.

### Functionality of PSI itself is impaired in the background of NDH-1 absence and multiple stresses

**Figure 6:**
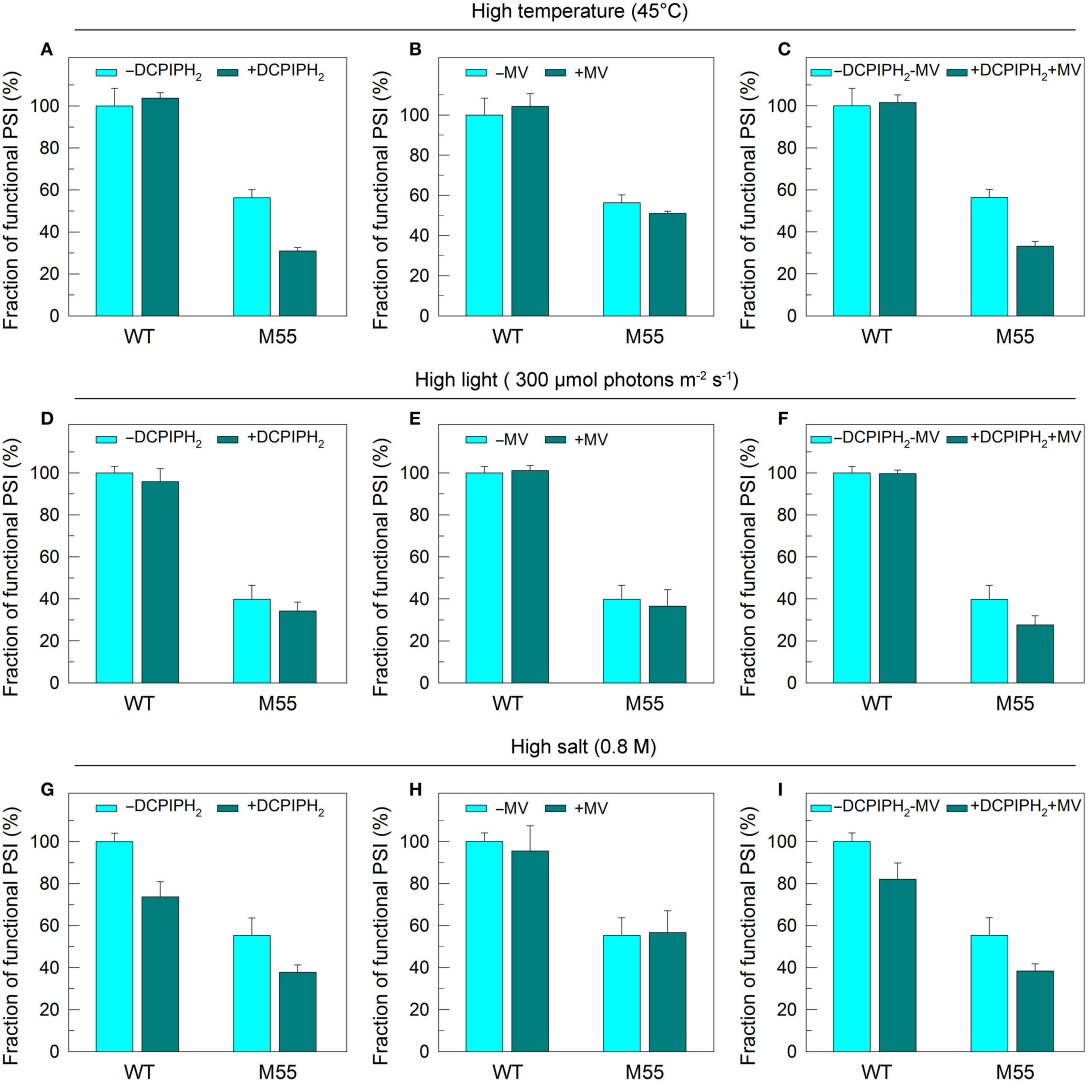
PSI functional fractions of WT and M55 under multiple stresses with PSI electron donor and/or acceptor. Cells were grown under normal salt conditions at 30°C and 40 μmol photons m^−2^ s^−1^ for 24 h and were transferred to 45°C for 12 h, 300 μmol photons m^−2^ s^−1^ for 36 h, or to 0.8 M NaCl for 12 h. The Chl *a* concentration of WT and M55 cells was adjusted to 20 μg mL^−1^. Prior to the *P*_m_ measurements, 200 μM 2,6-dichlorophenol-indophenol and 5 mM sodium ascorbate and/or 200 μM methyl viologen were added to the cell suspension, as shown in **(A–I)**. The functionality of PSI reaction centers was determined by the *P*_m_ parameter, expressed as a percentage of the WT (100%). The *P*_m_ value that corresponds to 100% is shown in the legend of Figure 1. Values are means ± *SD* (*n* = 5).

To test how NDH-1 affected PSI function under conditions of multiple stresses, we measured the PSI limitation at donor-side and acceptor-side as well as the functionality of PSI itself in WT and M55 strains. The reduced fractions of functional PSI under conditions of high temperature, high light and high salt were not recovered in M55 and were even slightly decreased by the addition of an exogenous PSI electron donor and/or acceptor to the cell suspension cultures of M55 (Figures 6A–I). Here, 2,6-dichlorophenolindophenol together with ascorbate (DCPIPH_2_) and methyl viologen (MV) were used as an exogenous electron donor (Mi et al., 1995) and acceptor (Takahashi and Katoh, 1984) for the PSI complex, respectively. Taking all these results together, we suggest that the impairment of PSI function in the absence of NDH-1 but the presence of multiple stresses is a result of impaired functionality of PSI itself.

DCMU addition and multiple stress treatments have a similar damage effect on PSI function in the absence of NDH-1 (see Figures 1, 2, 4) possibly because of suppressed PSII activity (Figures 1–3). To see whether the impairment of PSI function in DCMU-treated M55 cells also resulted from impaired functionality of PSI itself, we monitored the fractions of functional PSI in DCMU-treated cells of WT and M55 with or without DCPIPH_2_ and/or MV. Unexpectedly, the reduced fractions of functional PSI were completely recovered in M55 by the addition of DCPIPH_2_ with or without MV to the cell suspension cultures of M55 (Figures 4B,D), although they were still not recovered in M55 by the addition of only MV to the cell suspension cultures of M55 (Figure 4C). Therefore, the impairment of PSI function in the absence of NDH-1 but the presence of DCMU is a result of donor-side limitation of PSI. Based on the above results, we suggest that DCMU addition and multiple stress treatments have a similar damage effect on PSI function in the absence of NDH-1 but their impaired targets are different.

**Figure 7:**
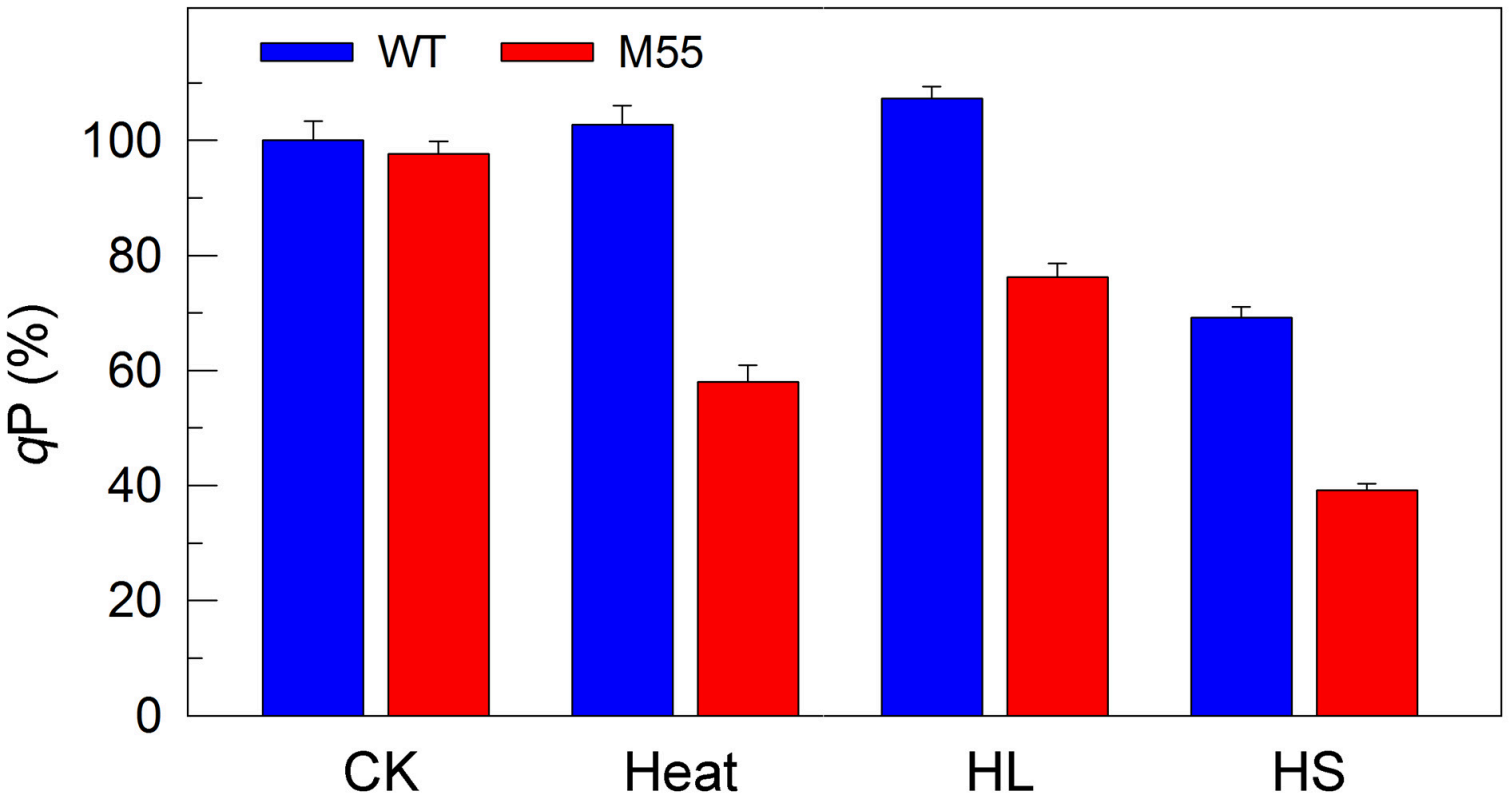
The ratio of the oxidized PQ pool (*q*P) of WT and M55 under multiple stresses. Cells were grown under normal salt conditions at 30°C and 40 μmol photons m^−2^ s^−1^ for 24 h and were transferred to 45°C for 12 h, 300 μmol photons m^−2^ s^−1^ for 36 h, or to 0.8 M NaCl for 12 h. Prior to the *q*P measurements, the Chl *a* concentration of WT and M55 cells was adjusted to 20 μg mL^−1^. The ratio of the oxidized PQ pool was determined by the *q*P parameter, expressed as a percentage of the WT (100%). The *q*P value that corresponds to 100% is 0.81 ± 0.03. Values are means ± *SD* (*n* = 5).

To obtain insights into the reason why DCMU addition and multiple stress treatments have different impaired targets in PSI, we measured the redox state of plastoquinone (PQ) pool in WT and M55 grown under conditions of multiple stresses. The fluorescence parameter *q*P can reflect the redox state of the PQ pool and its decrease is closely linked with reduction of the PQ pool (Misumi et al., 2016). Under conditions of high temperature, high light or high salt, the reduction level of the PQ pool in M55 was higher than that in WT as deduced from the *q*P values (Figure 7, right), regardless of similar redox state of the PQ pool under conditions of growth temperature (Figure 7, left). In addition, it is known that DCMU addition results in oxidation of the PQ pool via blocking the transfer of electrons from *Q*_A_ to *Q*_B_ in PSII (Trebst, 1980) and in the presence of DCMU, the oxidation level of the PQ pool in M55 might be higher than that in WT, because of absence of NDH-1-dependent cyclic and respiratory flows in M55 (Mi et al., 1992). Taking all these results together, we propose that multiple stresses and DCMU addition cause an opposite change in the redox state of the PQ pool, which may give a clue to the reason why they have different impaired targets in PSI.

### Absence of NDH-1 destabilizes the PSI complex under multiple stresses

**Figure 8:**
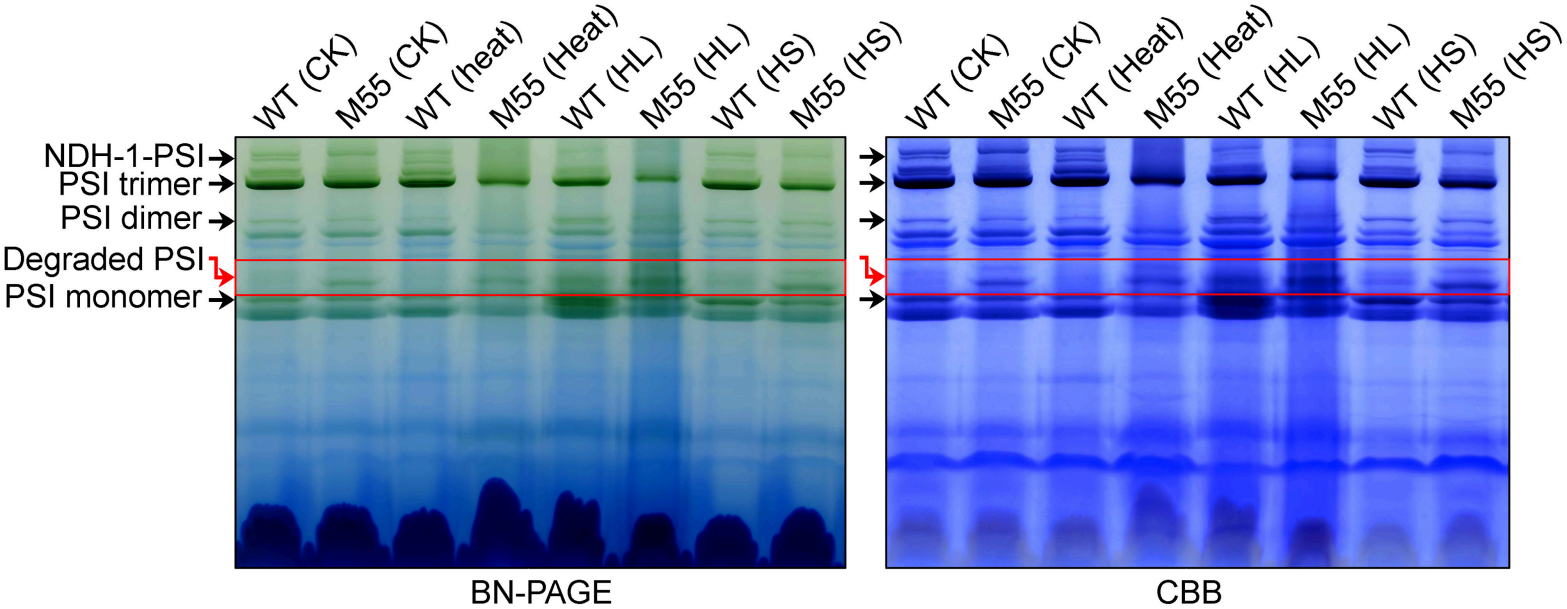
Accumulation of various PSI types under conditions of multiple stresses. Various PSI types isolated from the thylakoid membrane of WT and M55 cells were separated by BN-PAGE **(left)** and were stained by Coomassie Brilliant Blue (CBB; **right**). Black, and red arrows represent various PSI types and degraded PSI, respectively.

To understand why the functionality of PSI itself is impaired under conditions of high temperature, high light and high salt, we examined various types of PSI complexes in WT and M55 cells grown under these stress conditions. Under normal growth conditions, the absence of NDH-1 in M55 resulted in a slight destabilization of the PSI complexes compared to the WT (see red box in Figure 8; left), just like the results of previous studies (Gao et al., 2016a). Such destabilization became more evident under conditions of multiple stresses, as reflected by the relative contents of the PSI trimer, dimer and monomer (Figure 8; left); these differences between WT and M55 were clearer after staining with Coomassie Brilliant Blue (Figure 8; right). These results strongly suggest that absence of NDH-1 destabilized the PSI complex, specifically under multiple stresses, and consequently impaired the functionality of PSI itself.

## Discussion

Over the past decades, a significant achievement has been made in identifying the important role of cyanobacterial NDH-1 in protecting photosynthesis against environmental stresses, including protecting PSII and optimizing carbon assimilation via producing additional ATP and improving the ATP/NADPH ratio (Battchikova et al., 2011; Dai et al., 2013; Zhang et al., 2014; Zhao et al., 2014a,b, 2015; Gao et al., 2016a,b; Wang et al., 2016). The results of this study further indicate that cyanobacterial NDH-1 is important in maintaining the functionality of PSI itself (Figure 6), thereby guaranteeing a high level of PSI function under multiple stress conditions (Figures 1, 2). To our knowledge, this is the first study that reveals the contribution of NDH-1 to maintaining PSI functionality in cyanobacteria.

Recently, an NDH-1-PSI supercomplex was identified in the cyanobacterium *Synechocystis* 6803 (Gao et al., 2016a). We further found that absence of NDH-1 collapsed the supercomplex and resulted in a slight destabilization of the PSI complex, as deduced from the results of this study (Figure 8) and a previous study (Gao et al., 2016a) and that such destabilization became more evident under conditions of environmental stresses, as deduced from the results of this study (Figure 8) and a previous study (Zhao et al., 2014a). This finding may (1) explain why multiple stress treatments impairs the functionality of PSI itself, and (2) indicate that formation of the NDH-1-PSI supercomplex is important to keep a high level of PSI function under various stressful conditions.

Although the NDH-1-PSI supercomplex was also identified in angiosperms (Peng et al., 2008, 2009; Kouřil et al., 2014), the linker protein between NDH-1 and PSI as well as the component and function of NDH-1 were altered during evolution from cyanobacteria to higher plants. Stable formation of the supercomplex in higher plants needs two light-harvesting complex I (LHCI) proteins, Lhca5 and Lhca6, and in cyanobacteria needs a PSI-specific peripheral antenna, CpcG2-phycobilisome, but their homologs lack in each other (Peng et al., 2009; Gao et al., 2016a). In addition, NDH-1 included in the NDH-1-PSI supercomplex of cyanobacteria and higher plants has a similar L-shaped skeleton. Despite their similarity, a large number of NDH-1 subunits in higher plants, including ferredoxin-binding subcomplex subunits NdhT and NdhU and all the subunits of subcomplex B and lumen subcomplex, are absent in the cyanobacterial NDH-1 (for review, see Ma and Ogawa, 2015).

It is known that the NDH-1-PSI supercomplex mainly participates in NDH-CET (Peng et al., 2008, 2012; Gao et al., 2016a). NDH-CET can produce ATP via building a proton gradient across the thylakoid membrane, which is important for running the Calvin-Benson cycle under conditions of environmental stress; consequently, the presence of NDH-1 keeps a high PSI functional activity. In Arabidopsis, NDH-CET had no effect on PSI function even under conditions of fluctuating light, regardless of an important protecting role of PGR5-CET in PSI function under fluctuating light (Suorsa et al., 2012; Kono et al., 2014). Unexpectedly, in rice, NDH-CET plays an important contribution on PSI function, although the contribution is still minor compared with the PGR5-CET (Yamori et al., 2016). What's even more amazing is that NDH-1 seems to aid the antimycin A-sensitive PGR5-CET in Arabidopsis (Kou et al., 2015). By contrast, in *Synechocystis* 6803, NDH-CET is found to be important for PSI function under conditions of multiple stresses (Figures 1, 2) but PGR5-CET had little, if any, effect on PSI function even in the presence of DCMU (Figure 5). Based on the above results, we propose that the role of NDH-1 on PSI function has been altered during evolution from cyanobacteria to higher plants.

## Author contributions

WM: designed and supervised the experiments; JZ and FG: performed the experiments and analyzed the data; D-YF, WC, and WM: analyzed and interpreted the data and wrote the article.

### Conflict of interest statement

The authors declare that the research was conducted in the absence of any commercial or financial relationships that could be construed as a potential conflict of interest.

## Abbreviations

BN-PAGE: blue-native-PAGE
CBB: Coomassie Brilliant Blue
Chl: chlorophyll
DCMU, 3-(3, 4-dichlorophenyl)-1: 1-dimethylurea
*F*_v_/*F*_m_: maximal quantum yield of PSII
HL: high light
HS: high salt
M55: Δ*ndhB*
NDH-CET: NDH-1-dependent cyclic electron transport around PSI
MV: methyl viologen
PRG5: PROTON GRADIENT REGULATION 5
*P*_m_: the maximal P700 change
*q*P: the ratio of the oxidized PQ pool
*Synechocystis* 6803: *Synechocystis* sp. strain PCC 6803
WT: wild-type.
