# Supplementary material for: NDH-1 Is Important for Photosystem I Function of Synechocystis sp. Strain PCC 6803 under Environmental Stress Conditions
Source: Front Plant Sci. 2018 Jan 17;8:2183. doi: 10.3389/fpls.2017.02183 (PMC5776120; doi:10.3389/fpls.2017.02183)
Supplement: Supplementary file 2 [file Image1.pdf]

SUPPLEMENTARY MATERIAL

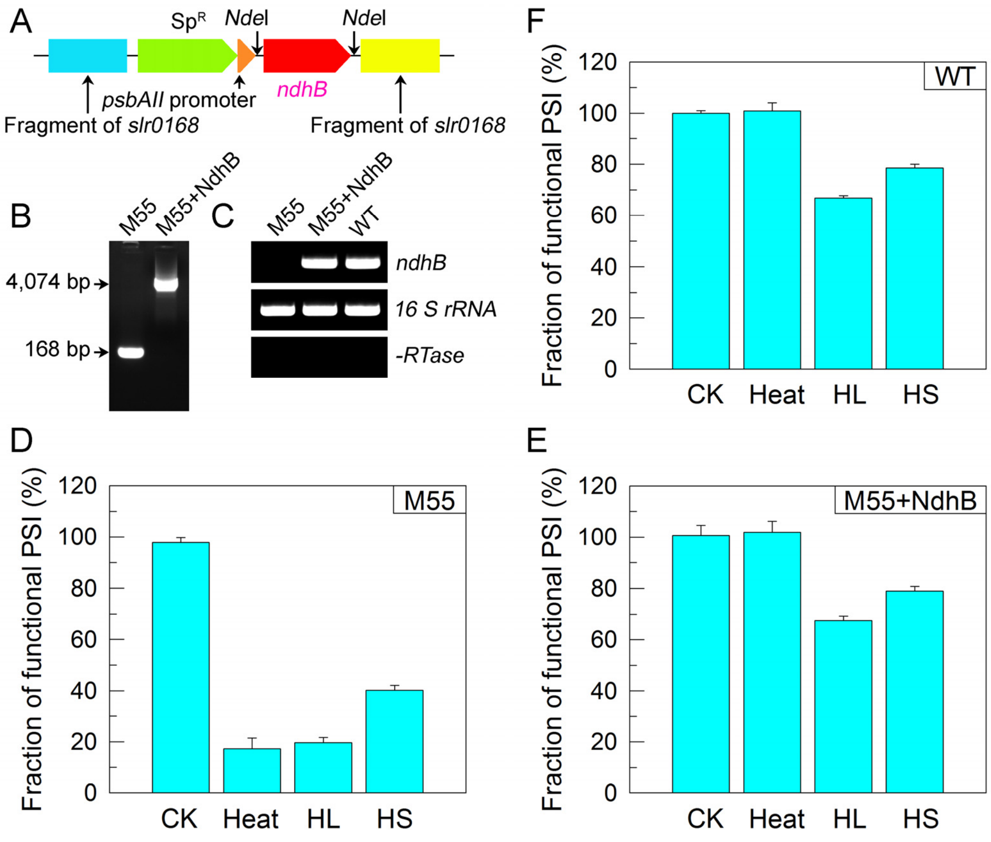

Supplementary Figure S1 Complementation of *ndhB* in M55 and its PSI functional fraction under conditions of multiple stresses. (A) Construction of P*psbAII*-*ndhB* expression vector used to generate the M55 complementation strain (M55+NdhB). **(B)** PCR segregation analysis of the M55+NdhB strain using the *ndhB*-C and *ndhB*-D primers (Supplementary Table S1). **(C)** Transcript levels of *ndhB* in the M55, M55+NdhB and WT strains. The transcript level of *16 S rRNA* in each sample is shown as a control. The absence of DNA contamination was confirmed by PCR without reverse transcriptase reaction. Cells of M55 **(D)**, M55+NdhB **(E)** and WT **(F)** grown under standard conditions for 24 h were transferred to 45°C for 18 h, to 300 μmol photons m^{-2} s^{-1} for 36 h or to 0.8 M NaCl for 12 h. Prior to the measurements, the Chl *a* concentration was adjusted to 20 μg mL^{-1}. PSI functionality was determined by the P_m parameter, expressed as percentage of the WT (100%). The P_m value that corresponded to 100% was shown in the legend of **Figure 1**.Values are means ± SD (n = 5). CK, control check; HL, high light; HS, high salt.
