# Supplementary material for: NDH-1 Is Important for Photosystem I Function of Synechocystis sp. Strain PCC 6803 under Environmental Stress Conditions
Source: Front Plant Sci. 2018 Jan 17;8:2183. doi: 10.3389/fpls.2017.02183 (PMC5776120; doi:10.3389/fpls.2017.02183)
Supplement: Supplementary file 3 [file Image2.pdf]

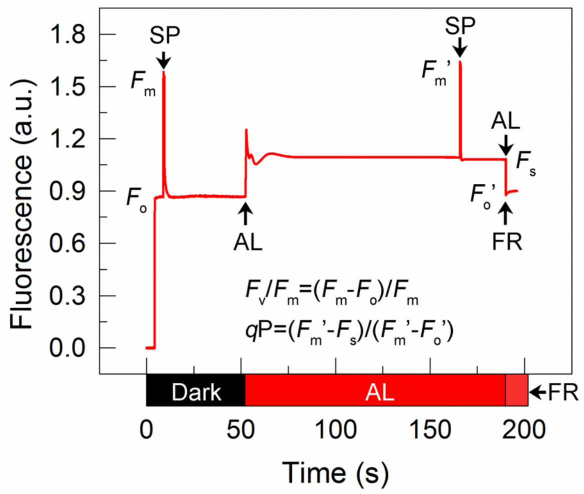

Supplementary Figure S2 A typical induction curve of Chl fluorescence in the cyanobacterium *Synechocystis* sp. strain PCC 6803 with saturation pulse analysis. Prior to the measurements, the concentration of Chl *a* was adjusted to 20 µg mL^{-1}. a.u. arbitrary units.
