# Supplementary material for: NDH-1 Is Important for Photosystem I Function of Synechocystis sp. Strain PCC 6803 under Environmental Stress Conditions
Source: Front Plant Sci. 2018 Jan 17;8:2183. doi: 10.3389/fpls.2017.02183 (PMC5776120; doi:10.3389/fpls.2017.02183)
Supplement: Supplementary file 4 [file Image3.pdf]

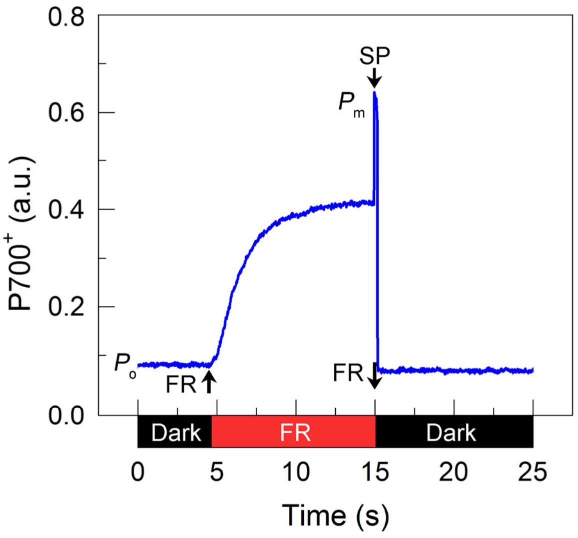

Supplementary Figure S3 A typical measuring method of P_m in the cyanobacterium *Synechocystis* sp. strain PCC 6803. Prior to the measurements, the concentration of Chl *a* was adjusted to 20 μg mL^{-1}. a.u., arbitrary units.
